# How to diagnose acute appendicitis: ultrasound first

**DOI:** 10.1007/s13244-016-0469-6

**Published:** 2016-02-16

**Authors:** Gerhard Mostbeck, E. Jane Adam, Michael Bachmann Nielsen, Michel Claudon, Dirk Clevert, Carlos Nicolau, Christiane Nyhsen, Catherine M. Owens

**Affiliations:** Department of Radiology, Wilhelminenspital, Montleartstr., 37 1160 Vienna, Austria; St George’s Hospital, Blackshaw Road, SW17 0QT London, UK; Department of Radiology, Rigshospitalet, Blegdamsvej 9, 2100 Copenhagen, Denmark; Children Hospital, University Hospital-Nancy Brabois, Rue du Morvan, 54511 Vandoeuvre Les Nancy Cedex, France; Munich University Hospital, Marchioninistraße. 15, 81377 München, Germany; Radiology Department, Hospital Clinic, Villarroel 170, 08036 Barcelona, Spain; Radiology Department, City Hospitals Sunderland FT, Kayll Road, Sunderland, SR4 7TP UK; Department of Radiology, Great Ormond Street, WC1N, 3JH London, UK

**Keywords:** Appendicitis, Ultrasound, Computed tomography, Magnetic resonance imaging, Diagnostic algorithm

## Abstract

Acute appendicitis (AA) is a common abdominal emergency with a lifetime prevalence of about 7 %. As the clinical diagnosis of AA remains a challenge to emergency physicians and surgeons, imaging modalities have gained major importance in the diagnostic work-up of patients with suspected AA in order to keep both the negative appendectomy rate and the perforation rate low. Introduced in 1986, graded-compression ultrasound (US) has well-established direct and indirect signs for diagnosing AA. In our opinion, US should be the first-line imaging modality, as graded-compression US has excellent specificity both in the paediatric and adult patient populations. As US sensitivity is limited, and non-diagnostic US examinations with non-visualization of the appendix are more a rule than an exception, diagnostic strategies and algorithms after non-diagnostic US should focus on clinical reassessment and complementary imaging with MRI/CT if indicated. Accordingly, both ionizing radiation to our patients and cost of pre-therapeutic diagnosis of AA will be low, with low negative appendectomy and perforation rates.

*Main Messages*

• *Ultrasound (US) should be the first imaging modality for diagnosing acute appendicitis (AA).*

• *Primary US for AA diagnosis will decrease ionizing radiation and cost.*

• *Sensitivity of US to diagnose AA is lower than of CT/MRI.*

• *Non-visualization of the appendix should lead to clinical reassessment.*

• *Complementary MRI or CT may be performed if diagnosis remains unclear.*

## Introduction

Acute appendicitis (AA) is a disease with a high prevalence, requiring rapid and accurate diagnosis to confirm or exclude perforation. It is the most common abdominal emergency and has a lifetime prevalence of about 7 % [1]. The clinical diagnosis remains difficult, both in the paediatric and adult population, as the presentation is often atypical [2]. Symptoms are frequently non-specific and overlap with various other diseases [3]. Despite all improvements in clinical and laboratory diagnosis and the publication of various scoring systems to guide clinical decision-making, the fundamental decision whether to operate or not remains challenging. In an ideal medical world, we would like to optimally diagnose and treat all patients with suspected AA without unnecessary appendectomies. As AA with perforation is associated with significant morbidity and an increase in mortality [2], there is broad agreement that high rates of negative appendectomies (around 15 %) have to be accepted in order to reduce the rate of perforation [2, 3]. A negative appendectomy might not only expose the patient to the risk of the surgical procedure. Recently, a higher risk of acute myocardial infarction related to surgical removal of the tonsils and appendix before age 20 has been reported [4]. Further studies are needed, as the authors point out, but subtle alterations in immune function following these operations may alter the cardiovascular risk [4].

Accordingly, the rapid and now widely used application of imaging methods in the diagnostic armamentarium for AA is demonstrated by an increasing number of publications, starting from the first report on compression ultrasound (US) by JB Puylaert in 1986 [5]. Multi-detector computed tomography (MDCT) is considered the gold standard technique to evaluate patients with suspected AA, because of its high sensitivity and specificity [2, 3]. Magnetic resonance imaging (MRI) has also shown high accuracy in the detection of AA, especially when radiation protection in children and in pregnant patients is of major importance [2, 3]. On the other hand, research focusing on various aspects of US imaging in the diagnoses of AA has gained major importance over recent years as radiation protection [6], broad availability and cost-effectiveness became increasingly important aspects of modern imaging techniques in the diagnosis of AA. Accordingly, this paper will focus primarily on the state of the art of US imaging in patients with a clinical suspicion of AA and will try to make a case for US as the first-line imaging modality in this clinical setting.

## Appendicitis: aetiology and demographics

In children and in adults, AA is a common emergency condition occurring at any age, but usually between 10 and 20 years [2, 7]. There is a male preponderance, with a male to female ratio of 1.4 to 1 [2, 7]. The overall lifetime risk is 6.7 % for females and 8.6 % for males in the USA [8]. We do not know the cause of AA, but there are probably many contributing factors. The primary cause is probably luminal obstruction, which may result from fecaliths, lymphoid hyperplasia, foreign bodies, parasites and primary neoplasms or metastasis (as detailed in [9]).

## Clinical diagnosis of appendicitis

### Clinical signs and symptoms

According to [2], AA might be called simple AA in the absence of gangrene, perforation or abscess around the inflamed appendix, or complicated AA when perforation, gangrene or periappendicular abscess are present. Abdominal pain is the primary presenting complaint, followed by vomiting with migration of the pain to the right iliac fossa, described first by J Murphy in 1904 [10]. However, this classical presentation is quite often absent, either due to variation in the anatomic position of the appendix or the age of the patient, with atypical presentations seen often in infants and elderly patients [2].

### Laboratory markers

A good review of laboratory markers for the diagnosis of AA is provided by DJ Shogilev et al. [3]. The degree of white blood cell elevation, the value of C-reactive protein, the proportion of polymorphonuclear cells, a history of fever and other factors have been studied extensively for the diagnosis of AA, but lack sufficient specificity either alone or in combination. On the contrary, the absence of all of these laboratory parameters can potentially rule out the diagnosis of AA [3].

### Scores

Many “clinical scoring systems” (CSS) have been developed to assist clinicians in appropriately stratifying a patient’s risk of having appendicitis. An excellent overview is provided by G Thompson [11]. As these scores are quite often implemented in the method section of studies on the diagnostic performance of imaging techniques in patients with a clinical suspicion of AA, knowledge of the most popular scores is mandatory. These are the Alvarado score, introduced by Alvarado in 1986 and sometimes referred as the MANTRELS score (acronym of the eight criteria), and the paediatric appendicitis score (PAS) or Samuel score, reported by Samuel in 2002 [11]. The Alvarado score has been reported in numerous studies in paediatric and adult patients with a suspicion of AA.

The Alvarado score was calculated retrospectively in a study population of 119 adults with a suspicion of AA and non-visualization of the appendix in an otherwise normal US examination, followed by computed tomography (CT) within 48 hours [12]. No patient (n = 49) with an Alvarado score ≤3 had appendicitis, compared to 17 % (12/70) patients with an Alvarado score ≥4 [12]. The authors conclude that patients with a non-visualized appendix with an otherwise normal US examination and an Alvarado score ≤3 do not benefit from a CT study. In a paediatric study population with a suspicion for AA, US was combined with a clinical assessment using the PAS [13]. The negative predictive value (NPV) of US decreased with increasing PAS-based risk assessment. The authors recommend serial US examinations or further imaging when there is discordance between US results and the clinical assessment by the PAS score [13]. However, in clinical practice, these scores are used in only a few centres (1 out of 83) [14].

### Novel markers

Modern markers like interleukin 6, serum amyloid A, rinoleukograms, Calprotectin and others have been studied as diagnostic tools in AA [3]. The power of these studies is considered limited in clinical practice to date. For more details see [3].

## When to use imaging

It is crucial to avoid two potential situations in patients with suspected AA: (1) any delay in diagnosis and subsequent perforation of the appendix; (2) an unnecessary appendectomy. There is agreement that imaging techniques improve both of these clinical scenarios, due to the potential for early diagnosis and the high sensitivities (CT, MRI) and specificities (US, CT, MRI) of these techniques [2, 7, 9]. A recent study demonstrated that increased use of pre-operative imaging in patients with AA resulted in a cost-effective way to decrease the negative appendectomy rate (NAR) [15].

### Ultrasound

#### Real-time compression ultrasound

**Fig. 1:**
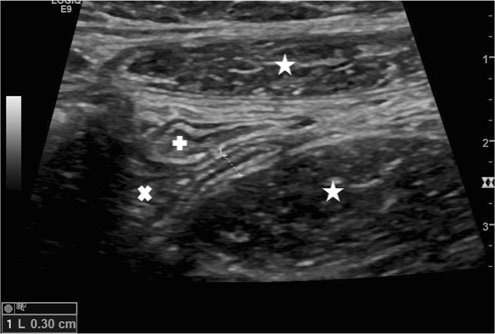
Longitudinal real-time US scan of a normal appendix. Diameter 0.3 cm. ** psoas muscle, * rectus muscle, x caecum, + terminal ileum

**Fig. 2:**
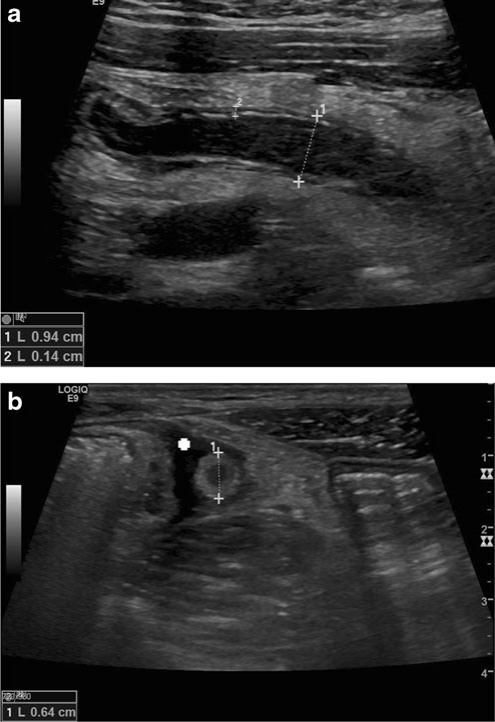
Longitudinal (**a**) and transverse (**b**) real-time US scan of acute appendicitis with thickening of the wall (*crosses 2*), target–sign, diameter > 6 mm (*crosses 1*) and free fluid surrounding the appendix (+)

Real-time compression US was first introduced by Puylaert in 1986 [5, 16]. Over the last 30 years, this technique has been extensively studied and improved (Figs. 1 and 2). Although the development of US technique has led to dramatic improvements in contrast, spatial and temporal resolution, US examination technique and US signs of appendicitis in real-time US have undergone only slight evolution. Graded-compression US is performed in a step-wise approach and aims to optimize visualization of the appendix [7, 9]. Recently, it has been shown that the diameter of the normal appendix (mean anteroposterior diameter 4.4 ± 0.9 mm, mean transverse diameter 5.1 ± 1.0 mm) does not change with age and is normally distributed in children [17]. To date, there are only few reports on the use of US elastography techniques in diagnosing AA [18, 19]. The same holds true for contrast-enhanced US (CEUS) [20, 21]. Besides, case reports in the largest series of 50 patients with suspected acute AA, L Incesu et al. [20] scored hyperemia in the wall of the appendix and prominent peripheral vascularity as seen by CEUS positive for AA. Direct and indirect US, Doppler and CEUS signs of AA both in the paediatric and adult patient are summarized in Tables 1 and 2.

**Table 1:** Alvarado score (score ≥7 = high-risk for appendicitis) and paediatric appendicitis score (Samuel score; adopted according to G. Thompson [11]). RLQ right lower quadrant of the abdomen

Alvarado score (MANTRELS)		Paediatric appendicitis score (Samuel score)	
Diagnostic criteria	Value	Diagnostic criteria	Value
Migration pain to RLQ	1	Migration pain to RLQ	1
Anorexia/acetone in urine	1	Anorexia	1
Nausea–vomiting	1	Nausea/emesis	1
Tenderness in RLQ	2	Tenderness in RLQ	2
Rebound pain	1	Cough/percussion tenderness	2
Temperature ≥ 37.3 °C	1	Pyrexia (not defined)	1
Leucocytosis (>10 x 10^3^/L	2	Leucocytosis (> 10x10^3^/L)	1
Leucocyte shift to left (>75 %)	1	Neutrophilia	1
Total score	10	Total score	10

**Table 2:** Direct and indirect (secondary) signs of acute appendicitis in graded-compression, real-time US, colour Doppler and contrast-enhanced US (CEUS; adopted according to references 7, 9, 20 and 21)

Real-time US signs of acute appendicitis	
Direct signs	Indirect signs
Non-compressibility of the appendix Perforation: appendix might be compressible	Free fluid surrounding appendix
Diameter of the appendix > 6 mm	Local abscess formation
Single wall thickness ≥ 3 mm	Increased echogenicity of local mesenteric fat
Target sign: Hypoechoic fluid-filled lumen Hyperechoic mucosa/submucosa Hypoechoic muscularis layer	Enlarged local mesenteric lymph nodes
Appendicolith: hyperechoic with posterior shadowing	Thickening of the peritoneum
Colour Doppler and contrast-enhanced US: Hypervascularity in early stages of AA Hypo- to avascularity in abscess and necrosis	Signs of secondary small bowel obstruction

In 2015, Trout et al. [22] reported on a three-category interpretative scheme of US-measured appendiceal diameters in the US diagnosis of AA in children. From 641 US reports (181/641 patients with AA, that is 28.2 %), data were used to generate a logistic predictive model to define negative, equivocal and positive categories for the diagnosis of AA [22]. Using cut-off diameters to define 3 categories (diameter ≤6 mm, > 6 mm to 8 mm, > 8 mm), AA was present in these categories in 2.6 %, 65 % and 96 %, respectively. The authors concluded that this three-category interpretative scheme provides higher accuracy in the diagnosis of AA than traditional binary cut-offs of 6 mm [22].

#### Results of US studies

In 2007 a systematic review including 9121 patients of 25 studies reported a sensitivity of 83.7 %, a specificity of 95.9 %, an accuracy of 92.2 %, a positive predictive value (PPV) of 89.8 % and an NPV of 93.2 % for the US diagnosis of AA [23]. The overall pooled estimates for the diagnostic value of CT were: sensitivity 93.4 %, specificity 93.3 %, accuracy 93.4 %, PPV 90.3 % and NPV 95.5 % [23]. For good-quality studies (five studies) comparing CT and US in the same population, CT was more sensitive (88.4 % vs 76 %) and a little bit more specific (90.4 % vs 89.4 %) than US [23]. In a recent review of the literature, there was an extremely variable diagnostic accuracy of US with sensitivities ranging from 44 % to 100 % and specificities ranging from 47 % to 100 % [24]. In a recent meta-analysis with head to head comparison studies of CT and graded compression US, CT had a better test performance than US [25]. Prevalence of AA in this meta-analysis was high (50 %, range 13 % to 77 %). One should not forget that post-test probabilities are markedly decreased when the pre-test probability is low, as has been demonstrated in this study [25].

### What is a non-diagnostic US examination?

In the early days of US for the diagnosis of appendicitis, it was clearly stated that US diagnosis relies on the “direct” visibility of the appendix and on “indirect” signs for local inflammation [16]. According to this paradigm, US examinations might be false–negative (a) if the inflamed appendix is overlooked; (b) if the inflamed appendix is overlooked and other abnormalities are erroneously considered responsible for the symptoms (e.g. ovarian cyst); (c) if the inflamed appendix is visualized but is considered not inflamed or is not recognized as the appendix [16]. A recent study demonstrated that greater abdominal wall thickness and a lower pain score were statistically associated with false–negative US examinations [26]. However, over recent years, various studies supported the hypothesis that a non-diagnostic US study (without US visibility of the appendix) might have a high NPV to rule out AA in specific patient populations and in specific clinical settings [27–32].

#### Visualization of the appendix

It seems quite obvious that body mass, thickness of the body wall and local pain might be factors responsible for excellent or absent visualization of the appendix by compression US. However, study results here are somewhat conflicting and inconsistent [33, 34]. Value of a “negative” US examination.

In a paediatric patient population, a retrospective chart review and outcome analysis was performed between 2004 and 2013 [27]. Out of 1383 US examinations, 876 (63 %) were non-diagnostic for AA and of these, 777 due to non-visualization of the appendix. Of these, 671 were considered ultimately true negatives, leading to a NPV of 86 %. Based on these results, the authors conclude that children with a non-diagnostic US study and without leucocytosis may safely avoid further diagnostic workup for suspected AA [27]. Similar results have been reported for 526 out of 968 children with “incomplete” visualized appendices and a clinical suspicion of AA [28]. Of these 526 patients, 59 % were discharged, 11 % were sent to the operating room and 30 % were admitted to the hospital for further observation [28]. Ultimately, 15.6 % of children with incomplete visualization of the appendix have been diagnosed with AA, but only 0.3 % of the children discharged home were ultimately diagnosed with AA [28].

In another retrospective study in children, the appendix could not be visualized in 241 studies (38 %) [29]. The authors analysed secondary US signs, like large amounts of free intrabdominal fluid, phlegmon and pericaecal inflammatory fat changes [29]. In this study, these secondary signs had a high specificity (98 % – 100 %) for the diagnosis of AA [29].

Shah et al. [30] reported on the type and incidence of disorders revealed by short-interval CT after non-visualization of the appendix by US in patients with suspected AA and otherwise normal US findings. In this retrospective analysis, 250 of 318 patients (78.6 %) revealed normal findings on CT. Appendicitis was diagnosed by CT in 16.4 % of patients, and another 5 % of the study population had an “important” diagnosis, necessitating urgent surgical therapy in only 0.6 % [30]. Based on their data, the authors argued for the development of clinical triage methods that differentiate patients who are likely to benefit from short-interval CT [30]. Another recent study reported little benefit to additional CT when the clinical appendicitis score was <6, and US did not show the appendix or evidence of inflammation [31]. Other investigators [32] have shown the safety of discharge of children with non-visualization of the appendix on US. Less than 0.3 % of these discharged children had a final diagnosis of AA [32].

#### Diagnostic algorithms

In order to keep radiation dose and financial cost low, various algorithms have been recently published for the work-up of a patient with suspected AA.

Ramajaran et al. [35] report their retrospective outcomes analysis for suspected AA, at a children’s hospital, over a six-year period. Their pathway established US as the initial imaging modality, and CT was recommended only if US was “equivocal”. 407 (60 %) of 680 study patients followed the pathway. Of these, 200 patients were managed definitively without CT [35]. The NAR was 7 % [35].

A protocol was implemented in another children’s hospital with an algorithm relying on 24-hour US as the primary imaging study in children with suspected AA [36]. The number of CT examinations per appendectomy decreased from 42 % before, to 30 % after implementation of the protocol, leading to reduced radiation exposure and imaging charges [36].

Another study reported on a set of clinical features that can rule out appendicitis in patients with suspected AA and non-diagnostic US results [37]. Patients were discharged after inconclusive US if less than two predictors were present: male sex, migration of pain to right lower quadrant, vomiting and leucocyte count >12.0 x 10^9^/l and re-evaluated the next day. The implemented clinical decision rule reduced the probability of AA in a large subgroup of patients with negative or inconclusive US results [37].

What if not only one initial US examination is performed, and an initial equivocal US examination is followed by clinical reassessment, a short-interval US and surgical consultation? This algorithm was studied prospectively in 294 children with a suspicion of AA and a baseline paediatric appendicitis score ≥2 [38]. Of the 111 children with AA, 108 were identified without use of CT. The short-interval US confirmed or ruled out AA in 22 of 40 children with equivocal initial US. The authors conclude that their approach is most useful in children with an equivocal initial US [38].

For a period from 2008 to 2013, another study reported a significant increase in the use of “US first” amongst 3353 children in Washington [39] following national recommendations to use US for the diagnosis of AA when possible. However, over 40 % of children were examined by CT. Of these, 35 % of all CT examinations were performed after an indeterminate US examination [39].

Van Atta et al. [40] have shown that implementation of an imaging protocol using US as the primary modality to evaluate paediatric patients with suspected AA leads to a decrease of CT utilization because 326 of 512 patients (63 %) did not require a CT examination.

#### Suggestions for optimal reporting

Another approach to improve US in the diagnosis of AA is standardized structured reporting. Instead of using a “binary” reporting system (positive–negative for AA), Larson DB et al. [41] introduced a five-category interpretative scheme (1–3: positive, intermediate likelihood or negative US examination when the appendix was visualized, respectivel; 4–5: secondary signs present or absent, when the appendix was not visualized). Accuracy of the five-category scheme was 97 %, compared to 94 % using a binary scheme [41].

#### Computed tomography

**Fig. 3:**
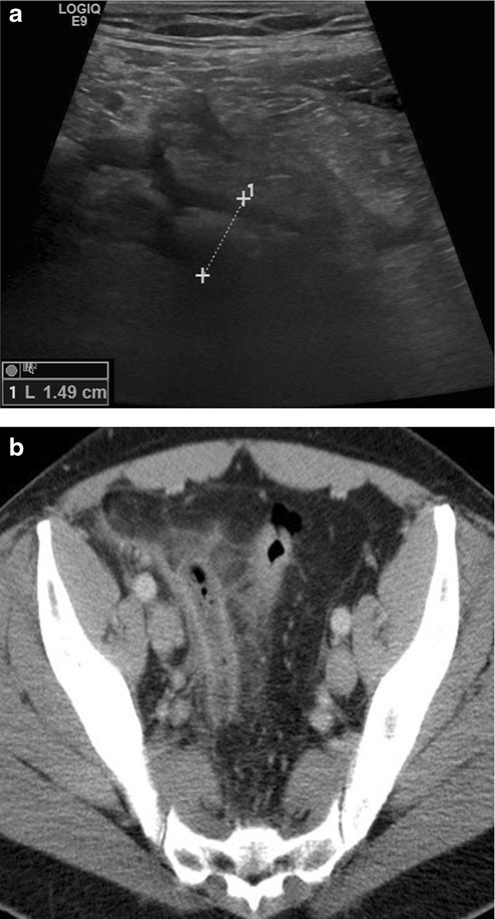
US and CT in acute appendicitis. 45-year-old male patient with pain in the right lower quadrant and increased inflammation parameters (white blood cell count and C-reactive protein elevation). **a** US real-time scan: local pain in combination with some fluid and thickened appendix, only seen in part (between *crosses*). **b** contrast-enhanced CT: thickened appendix, mesenteric infiltration around the appendix, inflammatory thickening of the sigmoid colon

There are many studies focusing on the examination technique of CT (with/without oral/rectal/intravenous contrast) and on optimal reconstruction parameters for the diagnosis of AA, which is beyond the scope of this article. However, there are convincing results on the high accuracy of CT for the diagnosis of AA (Fig. 3). A recent meta-analysis [42] included 9330 patients published in 28 studies and reported a significant difference in the NAR, from 16.7 % when using clinical evaluation without imaging compared to 8.7 % with use of CT. In addition, the NAR decreased from the pre-CT era to the post-CT era (21.5 % to 10 %) [42]. In 2011, MDCT showed a sensitivity of 98.5 % and a specificity of 98 % for the diagnosis of AA in 2871 patients [43]. Another meta-analysis included 4341 patients (children and adults) from 31 studies and reported a pooled sensitivity and specificity for diagnosis of AA in children of 88 % and 94 %, respectively, for US studies and 94 % and 95 %, respectively, for CT studies. Pooled sensitivity and specificity for diagnosis of AA in adults were 83 % and 93 %, respectively, for US studies, and 94 % and 94 %, respectively, for CT studies [44]. Recently, it was shown that low-dose CT is not inferior to standard-dose CT (median dose–length product 116 mGy · cm vs 521 mGy · cm) with regards to NAR [45]. When conditional CT (a CT study after a negative or inconclusive US examination) is used compared to an immediate CT strategy in an adult patient population with a suspicion of AA, these conditional CT exams correctly identify as many patients with AA as an immediate CT strategy, but only half of the number of CTs is needed [46].

On the other hand, if CT findings are in the favour of “probably not appendicitis” or “equivocal appendix”, then US re-evaluation of these patients may be helpful [47]. In this CT study of 869 patients with suspected AA, 71 (8 %) had equivocal appendicitis findings and 63 (7 %) were diagnosed as probably not appendicitis [47], clearly demonstrating that CT findings, in daily clinical routine, cannot be always reported in a binary “yes–no” category either.

#### Magnetic resonance imaging

**Fig. 4:**
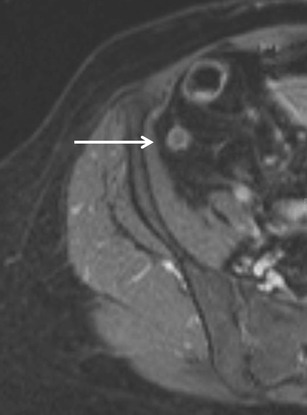
T1-weighted, fat-suppressed axial MRI after intravenous MRI contrast (gadoterate) in acute appendicitis: thickened appendix with Gd enhancement, minimal periappendiceal stranding

MRI is gaining relevance as a problem-solving technique or when US is inconclusive, mainly in populations where radiation protection is of special importance. In 33 pregnant patients with a clinical suspicion of AA, US was compared to MRI [48]. Only 5 of these 33 patients had pathologically-proven appendicitis. When the appendix was visualized on US and on MRI, sensitivity and specificity for MRI were both 100 % and for US, they were 50 % and 100 %, respectively. This is a nice example for a study that is limited by a small study population and a low prevalence of the disease to be studied [48]. Recently, it has been shown that gadolinium-enhanced images (Fig. 4) and T2-weighted images are most helpful in the assessment of AA in the paediatric population [49]. Another recent publication demonstrated that in children with suspected AA, strategies with MRI (MRI only, conditional MRI after negative or inconclusive US) had a higher sensitivity for AA compared with an US-only strategy [50]. In children with suspected AA, a radiation-free diagnostic imaging algorithm of US first selectively followed by MRI has been shown to be feasible and performed excellent compared to CT in terms of NAR, perforation rate or length of hospital stay [51]. Similar results with an excellent MRI sensitivity of 100 % and specificity of 96 % have been reported by others in paediatric patients after inconclusive US [52].

## Why use US as first-line imaging modality?

In conclusion, the studies and reports detailed above give an overview of the persistent difficulties in the clinical diagnosis of AA in paediatric and adult patients, the usefulness of various clinical scores (which are not commonly used in routine practice) and recent developments of modern imaging techniques focusing on US imaging. To date, US imaging for suspected AA is performed world-wide by radiologists and many physicians of other medical subspecialties, with or without the support of sonographers.

Paediatric patient population: It is the opinion of the ESR working group on US that graded-compression US should be the first-line imaging modality in paediatric patients with suspected AA. Direct and indirect US signs of AA are well established, as is the examination technique itself. We recognize that US has, compared to CT and MRI, lower sensitivity for the diagnosis of AA [25]. The 2011 ACR Appropriateness Criteria® for right lower quadrant pain — suspected appendicitis — state that “for adult patients with clinical signs of AA the sensitivity and specificity of CT are greater than those of US, but that in paediatric patients, the sensitivity and specificity of graded-compression US can approach those of CT, without the use of ionizing radiation” [53].

Adult patient population: In the adult and especially in the elderly patient, where the sensitivity of US might be limited and important differential diagnoses have to be considered, CT might be used as the first-line imaging technique. When US is the suggested first line for all patients, consideration should be made that this may be difficult in many countries and hospitals due to outsourcing of radiologic services at night, due to the limited availability of US-experienced radiologists, physicians or sonographers 24/7, or due to a combination of all of these factors.

Regarding the patient with nonvisualization of the appendix itself on US, or other reasons for non-diagnostic US examinations in this setting, careful clinical re-assessment of the patient is recommended and complementary imaging should follow, if necessary. Depending on the local environment and expertise, this might be a second US examination, an MRI examination when radiation protection is mandatory (paediatric and pregnant patients) or a CT examination where diagnostic criteria and high accuracy are well-established. It has been demonstrated in a recent meta-analysis [54] that an imaging protocol using US as a first-line imaging tool, followed by CT, offers significant cost savings over a CT-only protocol, and avoids radiation exposure. In a Markov-based decision model of paediatric appendicitis, the most cost-effective method of imaging children with suspected AA was to start with US and follow each negative US examination with a CT examination [55]. However, the economic and radiation burden considerations have to be translated to the specific healthcare system and cannot be transformed to all clinical and geographic settings.

## Abbreviations

AA: acute appendicitis
US: Ultrasound
CEUS: contrast-enhanced US
CT: computed tomography
MRI: magnetic resonance imaging
NAR: negative appendectomy rate
